# Study of the Physico-Mechanical Properties and Oxygen Permeability of Bacterial-Cellulose-Based Conduits

**DOI:** 10.3390/polym17152123

**Published:** 2025-07-31

**Authors:** Marina V. Parchaykina, Mikhail A. Baykov, Elvira S. Revina, Mikhail V. Shchankin, Viktor V. Revin

**Affiliations:** Department of Biotechnology and Biochemistry, National Research Ogarev Mordovia State University, 430005 Saransk, Russia; mikhail.baikov.a@yandex.ru (M.A.B.); rewina.elvira.s@yandex.ru (E.S.R.); schankinmv@mail.ru (M.V.S.); revinvv2010@yandex.ru (V.V.R.)

**Keywords:** BC, IR-spectroscopy, SEM, oxygen permeability, biomedicine, conduits

## Abstract

The article is devoted to the study of the physico-mechanical properties and oxygen permeability of the examined conduits based on bacterial cellulose (BC) obtained using the *Komagataeibacter sucrofermentans* B-11267 strain. BC is considered a promising material for regenerative biomedicine. The chemical structure, crystallinity degree and porosity of BC-based conduits were characterized by means of infrared spectroscopy (IR-spectroscopy), scanning electron microscopy (SEM) and atomic-force microscopy (AFM). Both the Young’s modulus and determined tension showed the high strength of the obtained conduits. Their oxygen permeability exceeded the values for the existing analogues, and lack of cytotoxicity indicated biocompatibility, confirming that BC-based conduits may be used for biomedical purposes.

## 1. Introduction

Currently, one of the most urgent and unsolved problems of regenerative biomedicine is the complete recovery of normal functioning of damaged tissues and organs, since, in many cases, there is partial regeneration accompanied by disablement and fatality. In particular, the problem of somatic nerve regeneration has attracted the attention of researchers, since there is the pressing clinical need to develop technology that is capable of promoting the recovery of nerve conductors and their functional activity improvement. Despite significant advances in surgical approaches, the regeneration of motor and sensory functions after injury is frequently unsatisfactory [1]. One solution is to develop biopolymer constructions that accelerate the recovery processes in damaged cell structures, or act as carriers for the targeted delivery of drugs and biologically active substances to the injury [2].

Natural polymers, also called biopolymers, appear to be highly promising for use in this function [3,4], since they represent a group of highly efficient materials, are widely available, and have no side effects [5,6]. Moreover, they exhibit high biocompatibility with the extracellular matrix [7], resulting in their low cytotoxicity [8,9]. Among various natural and synthetic materials, BC attracts the greatest attention in biomedical research [10,11]. BC fibers are generally several nanometers in diameter and several µm in length; these are typically gathered in a highly ordered 3D porous network. BC porosity is the governing factor [5,12,13] providing high permeability for gases and liquids, which is the necessary condition for developing conduits. The major characteristics of BC fibers are as follows: high nutrient metabolism, provide oxygen, and discharge exudate. As a result, BC-based conduits should have higher efficacy compared with the existing analogues [14,15,16]. The ultrafine structure of BC fibers enables the production of material with a low density but high mechanical strength [17,18]. Additionally, BC’s chemical modification ability makes it universally applicable [19]. For instance, carboxylation or phosphorylation can enhance BC biological activity or BC interactions with other biological objects, further extending their applicability in biomedicine [20,21,22].

In static conditions, BC forms on nutritional media, at the air–liquid interface, generally forming a thick and dense membrane on the surface. The method is preferable for obtaining the BC-based matrix. Agitated culture conditions include medium stirring or shaking; these help disperse bacteria throughout the liquid, resulting in the formation of agglomerates of various shapes [23]. Although agitated fermentation can improve the dissolved oxygen content by shaking or stirring compared with static fermentation, cells are prone to mutate during mechanical stirring of the culture; this leads to their loss of ability to produce BC [19]. Moreover, the culture under static conditions is more beneficial for producing conduits, since this culture allows cellulose to form with minimal distortions, preserving its inner structural properties. The latest advances in BC production include the gene engineering of bacteria to increase output and the quality of the produced cellulose. According to data from the literature, using G. Xylinus with knockout membrane glucose dehydrogenase can increase BC productivity by up to 40%. Researchers are also studying the use of different substrates, particularly the waste products from biotechnological production, as nutritional medium components; the aim is to reduce the expense and increase BC output [24]. In particular, using the previously mentioned concentration of molasses, it is possible to obtain 1.5-fold more target product per 1 g of the raw material for the molasses medium [10].

**Figure 1:**
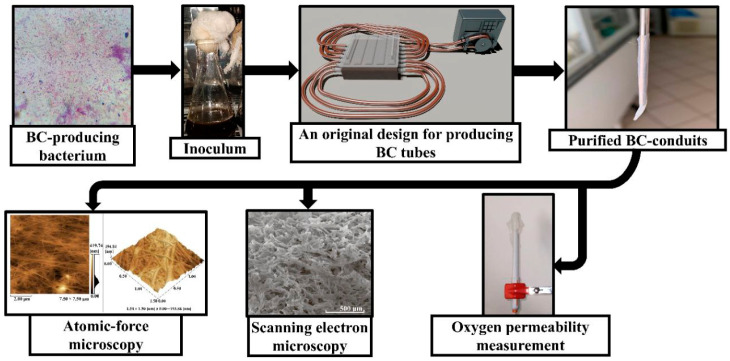
Scheme of production, physico-mechanical properties, and oxygen permeability investigation of BC-based conduits.

In the present study, we obtained BC-based conduits using the static culture of the *K. sucrofermentans* B-11267 strain on the medium with a molasses concentration 50 g/L. This is because at this particular molasses concentration, most BC was found to be produced within first three culture days; this is intended to be cost-efficient, and, on an industrial scale, can result in significant cost savings [10]. Generally, BC has a promising future in biomedicine, since its unique structural characteristics combined with the utmost mechanical strength and biocompatibility make the polymer an ideal candidate for different applications ranging from wound dressings to elaborated frames in tissue engineering [23,25]. Despite significant advances in BC-based conduit usage in tissue engineering, currently there are some problems remaining, such as providing sufficient oxygen to recover damaged cell structures, since oxygen is needed for metabolism and the functional activity recovery of tissues and organs; moreover, oxygen deficiency can cause damage to the native structure, including apoptosis and necrosis. Thus, the objective of the present research was to study the physico-mechanical properties and oxygen permeability of BC-based conduits for their application in regenerative medicine for the recovery of damaged cell structures (Figure 1).

## 2. Materials and Methods

### 2.1. BC-Based Conduits Production

In the present study, *Komagataeibacter sucrofermentans* B-11267 was used in the production of BC-based conduits. The *K. sucrofermentans* B-11267 strain was isolated from kombucha and deposited in the Russian National Collection of Industrial Microorganisms (VKPM) (registration number: B-11267) [26]. The strain was cultured in the media containing beet molasses concentrations of 50 g/L with a pH 4.5. The beet molasses used in the present study were obtained from Romodanovsky Sugar Factory (Mordovia, Russia). The beet molasses had the following composition (% by weight): dry matters—82.0; sucrose—50.0; total nitrogen—2.3. Prior to inoculation, the media were autoclaved at 121 °C for 20 min. The inoculum used for seeding nutrient media was prepared using the method previously described [27]. BC-based conduits were produced by fermenting *K. sucrofermentans* B-11267 in a container with two 200 cm silicone tubes, their internal diameter being 5 mm, using a molasses medium in a concentration of 50 g/L. The medium was inoculated with 10% (*v*/*v*) inoculums. For this purpose, 1350 mL of the liquid nutrient medium and 150 mL of the inoculum were added to the container under aseptic conditions. Each silicone tube contained 39 mL of the culture medium. The *K. sucrofermentans* B-11267 strains were cultured for 7 days, with double mixing of the nutrient medium in each tube for 10 min on days 1 and 2. For mixing, we used a peristaltic dosing pump LOIP LS-301 (LOIP, St. Petersburg, Russia) at a rotor speed of 200 rpm. At the end of the process, the nutrient medium and the resulting gel film were removed from the container, and 1500 mL of distilled water was added. To remove the silicone tubes from the tubular structures, we carried out a process similar to that for mixing the medium, resulting in pushing the biopolymer out of the tubes using a stream of water. The resulting structures were processed in order to remove the nutrient medium cells and components using the method previously described [27]. The obtained BC tubular structures were autoclaved at 121 °C for 20 min.

### 2.2. IR-Spectroscopy

Using the method described previously, the BC-based conduit samples were freeze-dried and crushed into powder, mixed with potassium bromide, and pressed into a small tablet; after that, they underwent analysis by the chosen method using an IR-spectrometer IR Prestige-21 (Shimadzu, Tokyo, Japan,) to examine absorption behavior. Each specimen was scanned in 32 replications, at resolution 4 cm^−1^ in the middle IR-range 4000–400 cm^−1^ [10].

### 2.3. Scanning Electron Microscopy

The microstructure of BC-based conduits was studied using a multifunctional raster electron microscope QuantaTM 3D 200i (FEI Company, Hillsboro, OR, USA).

### 2.4. Atomic-Force Microscopy

The surface morphology of the BC-based conduits was studied by AFM using a scanning probe microscope SPM 9600 (Shimadzu, Tokyo, Japan). The BC-based conduit samples were dried on a glass slide in the form of a thin film. A silicon nitride cantilever with a pyramidal probe, 2 nm in radius, was used. The scanning frequency varied from 0.6 to 1.0 Hz. The image resolution was 256 × 256.

### 2.5. X-Ray Diffraction

The crystallinity changes in the obtained BC-based conduits were assessed using a previously described X-ray diffraction (XRD) technique. This was carried out using an Empyrean X-ray diffractometer (PANalytical, Almelo, The Netherlands) and using filtered radiation of the copper anode (λ = 0.15418 nm, 40 kV, 30 mA) in the angular range from 10° to 60°. We also used a coordinate detector, Pixel 3D, in the linear scanning mode (255 points on a line), with a resolution of 0.013 degrees/line [27]. The obtained samples were freeze-dried using FreeZone Freeze Dry System (Labconco, Kansas City, MO, USA). The crystallinity index (CrI) was calculated from the ratio of the height of the 002 peak (I_002_, 2θ = 22.5°) and the height of the minimum (I_am_) between the 002 and 110 peaks (I_am_, 2θ = 18°) (Equation (1)).

CrI (%) = [(I_002_ − I_am_)/I_002_] × 100%. (1)

### 2.6. Strength and Tensile Measurement

To measure strength, the samples were cut into rectangles, 2 × 1 cm in size. The thickness was measured using the high-resolution automatic thickness gauge CHY-C2 Thickness Tester (Labthink, Jinan, China); this was carried out by recording the values for each of the samples in several places in order to calculate the average.

After cutting, the BC-based conduits were also assessed on a test machine, the PARAM^®^ XLW (PC) Auto Tensile Tester (Labthink, Jinan, China). The samples were fastened in the machine forceps and stretched until the samples ruptured at 50 mm × min^−1^.

We used the obtained data to calculate Young’s modulus, describing the material’s ability to resist deformity, compression, and tensile stretching, which was significant for the conduits. The calculations were made using the following formula [28].

(2) $$E=\frac{Fl}{S\Delta l},$$

where F is the force applied to rupture the sample, l—length, S—the sample square, and Δl—the sample’s tensile measurement prior to the rupture.

### 2.7. Oxygen Permeability Measurement

The oxygen permeability of the studied BC-based conduits was assessed on an oximeter DO210E using a sensor DO-957-Q (Fcombio, Zhengzhou, China) to examine both saturation and concentration measurement modes. The obtained BC-based conduits were cut into rectangles, 1 × 2 cm in size, for further assessment of the required characteristics, with accuracy of up to 2% or 0.3 mg/L of the chosen equipment. Each sample was tightly rested against the electrode membrane; after having made sure that the BC-based conduits were completely seated to the measuring section of the device, we recorded the value 15 min after starting the measurement. Between the measuring procedures, the samples were kept in cold (up to 4 °C) distilled water to prevent them from drying and developing pathogenic microbial flora.

### 2.8. MTT Assay

To determine cytotoxicity, using MTT assay, the preliminary obtained conduits were crushed on a laboratory homogenizer Bioprep-24 (Allsheng, Hangzhou, China) for 10 min to obtain the BC hydrogel with a hydraulic module at the ratio of 1:3 (i.e., 1 mL of water per 3 g of BC-based conduits). The human embryonic lung cells were cultured in DMEM supplemented with 10% fetal calf serum, penicillin, streptomycin, and glutamine (PanEco, Moscow, Russian Federation). The cells were harvested from the plates using 0.25% trypsin–EDTA solution (PanEco, Moscow, Russian Federation) and inoculated into a 96-well plate in a concentration of 10,000 cells per well followed by adding the hydrogel to the cells. The cytotoxic effect of the material was assessed using 3-(4,5-dimethyl-2-thiazolyl)-2,5-diphenyl-2H-tetrazolium bromide (MTT, Sigma Aldrich, St. Louis, MO, USA) in a concentration of 5 mg/mL according to the method described in [29]. The plates were incubated for 3.5 h until violet formazane crystals formed. After that, the crystals were dissolved with dimethylsulfoxide solution on a plate shaker for 20 min at 37 °C. Optical density was measured at a wavelength of 570 nm in comparison with the reference at 650 nm on a Varioscan Lux (MTT, Sigma Aldrich, St. Louis, MO, USA). The cell viability was determined as the ratio between the optical densities of the experimental and the control specimens and expressed as a percentage.

### 2.9. Statistical Analysis

All presented data are averages of at least 3 runs of experiments, performed with 10 replicates of the mean. Standard deviations of the mean were calculated using Microsoft Excel 2013 (Microsoft Corporation, Redmond, WA, USA). The obtained data were statistically analyzed by using two-way ANOVA followed by Tukey’s multiple comparison test for pairwise group comparisons, with a significance threshold of 5%.

## 3. Results and Discussion

BC is characterized by its unique mesh structure represented by thin nanosized fibers; their characteristics are 100 times lower than those from plant analogues. According to the previous studies, the structure and properties of BC depend on the strain used, the medium composition, and the culture method [10].

**Figure 2:**
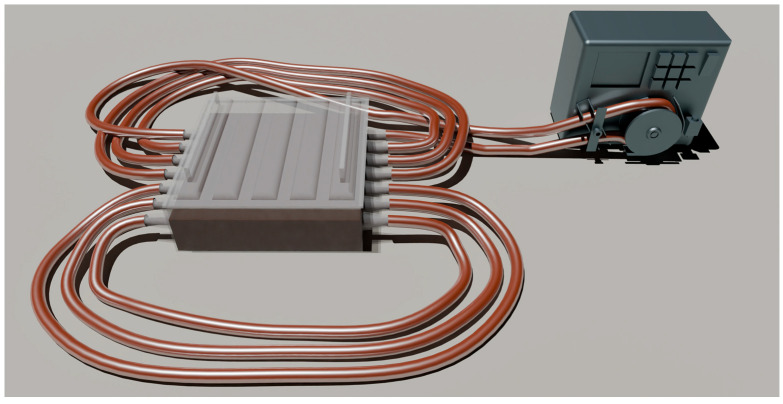
An original design for producing BC-based conduits.

The most significant properties of the conduits used in regenerative biomedicine are strength, oxygen permeability, and biocompatibility. We were the to develop a unique technology for producing bacterial-cellulose-based conduits in a molasses medium, exhibiting both high strength and oxygen permeability (Figure 2).

**Figure 3:**
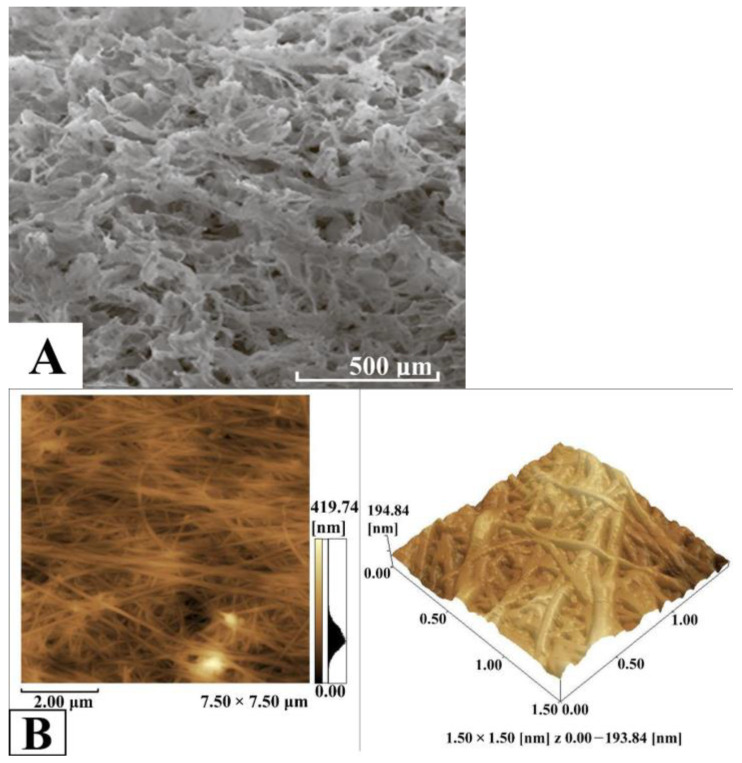
SEM-image of BC-based conduits (**A**), AFM-image of BC-based conduits (**B**).

The characteristics of BC-based conduits and their chemical structure were studied using SEM and IR spectrometry. The study of the structure and physicochemical properties of the obtained BC-based conduits is significant regarding the behavior of pharmaceuticals in the BC matrix and the kinetics of their release. When the pore size of BC-based conduits exceeds the molecular size of the drug, the diffusion coefficient is known to be able to relate to the material porosity and tortuosity. For composites with pore sizes close to the drug molecule size, or non-porous materials, drug diffusion coefficients decrease due to steric restriction of polymer chains. In such cases, the mean free volume of the drug molecules decreases, while hydrodynamic resistance to the drug grows. This results in an increase in the drug diffusion path length compared with the composites with pores, the sizes of which are much larger than in an encapsulated drug. The large surface area of the BC-based conduit pores obtained in the present study enabled us to load large quantities of the drug into the matrix. However, the microporous structure of BC-based conduits promotes slow release of the drug, resulting in a prolonged action of the material. In addition, the pore sizes of BC-based conduits are very important when using the materials in tissue engineering and regenerative medicine [30,31]. Three-dimensional scaffolds of BC provide a nearly ideal medium for cell growth and tissue development compared to 2D materials, which can provide just the surface growth. Thus, when developing biocomposite materials with the desired porosity, we can influence the drug release rate or cell growth and tissue development in cases of application in tissue engineering and regenerative medicine [15]. Figure 3 shows the three-dimensional mesh structure of the BC-based conduit fibrils with pores of different sizes corresponding to its typical structure formed by bacteria in biosynthesis. The photomicrography of the BC-based conduits obtained using SEM revealed microfibrillar bands (lines) ranging from 60 to 190 nm wide. The average thickness of BC-based conduit fibrils was 60–90 nm.

**Figure 4:**
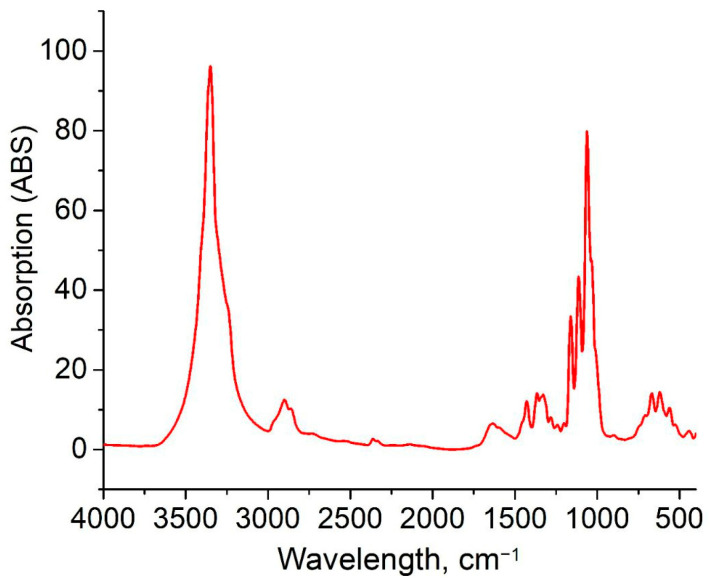
IR-spectrum of BC-based conduits.

The chemical structure of BC-based conduits was determined by the position and intensity of peaks in the IR-spectra; these are represented in Figure 4. The characteristic BC-based conduit absorption peaks were located at 3350 cm^−1^, and had several bands which were typical for cellulose: these were in the range of 1500–1235 cm^−1^ due to O-H bond extension, and at 2960 cm^−1^ due to C-H bond extension. The band at 1653.8 cm^−1^ was due to the deformation vibration of the absorbed water molecules. The band at 1068 cm^−1^ can be related to C-O-C ester functionality. According to Nelson and O’Connor, cellulose I is determined by the presence of a weak and wide band with its center at 891.59 cm^−1^ and an intense band with the center at 1424.18 cm^−1^ (CH_2_−) in the spectra of microbial cellulose samples [32]. Cellulose I is a form of cellulose synthesized by bacteria and consisting of parallel β-1,4-glucan chains. In cellulose I, there are two different crystalline phases—I_α_ and I_β_. BC crystallinity is one of its major properties, since the high crystallinity of BC provides both durability and resistance to fermentative degradation that is useful for long-term in vivo application. Crystal BC-based conduit regions are densely packed, reducing the accessibility of enzymes, and slowing down degradation, providing stable production in biological media [23]. The obtained BC-based conduits demonstrate the properties of small-sized crystallites, high crystallinity, and high cellulose I_α_ content.

**Figure 5:**
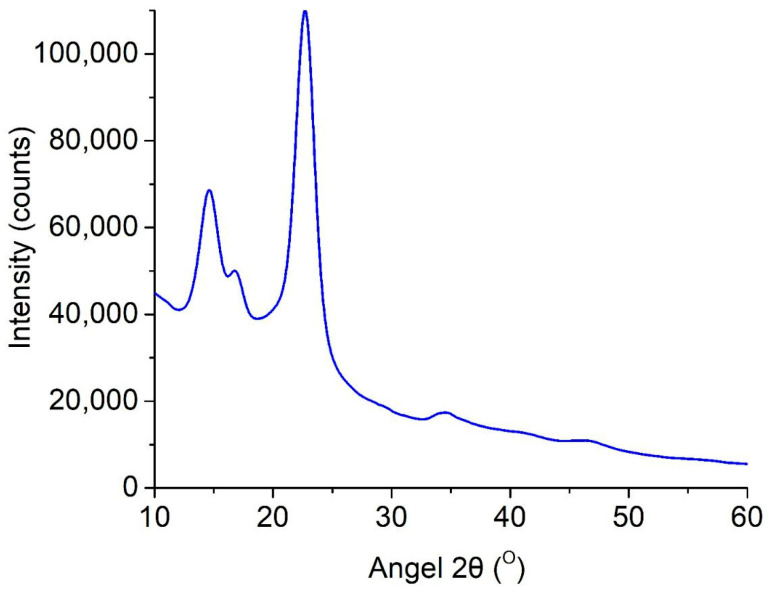
Diffractogram (reflection mode) of BC-based conduits.

Additionally, the medium composition and the used strain have a substantial impact on the degree of crystallinity as well. For example, using a medium based on paper industry waste and Acetobacter xylinum as a BC-producing microorganism, it is possible to obtain a material with a crystallinity index of 77.8–77.58%, which is slightly lower than the values we obtained [32,33]. To assess the crystallinity of the BC-based conduits, we used X-ray diffraction; this analysis resulted in a BC diffractogram, which is represented in Figure 5.

The BC-based conduit diffractogram was found to have three major peaks at 2θ—14.4°, 16.8°, and 22.5° corresponding to three crystallographic planes, (100), (010), and (110), respectively. The reflection intensity of the plane at 100 is higher than at 010, when a film is parallel to the X-ray, and the effect reverses in perpendicular orientation [34]. Moreover, there was strong uniplanarity related to the fact that cellulose tapes were primarily orientated parallel to the film surface during drying. The crystallinity index of BC-based conduits obtained on the HS medium was 79.7%.

The study also analyzed the physico-mechanical parameters of the obtained BC-based conduits. The films under study demonstrated high tensile capabilities—the maximum was 57.5%. Moreover, the tensile strength and Young’s modulus reached the values of 3.608 N and 8.952 MPa, respectively. Thus, compared to other methods of producing similar structures, the tubular structures we obtained showed significantly higher strength. In particular, Young’s modulus was 1 MPa higher, and the tensile strength was 1.418 N higher. The elasticity was 10% less than in similar static synthesis methods but more reproducible compared with the dynamic synthesis methods [35]. Table 1 represents the summary data.

To use BC as a conduit, it should characteristically exhibit high strength and oxygen permeability. In the junction of the electrode and the film under study, the oxygen permeability was 45.8 ± 0.01% compared with the upper layers of deionized water, according to which the instrument calibration was performed. Table 2 demonstrates the study findings.

The results of the BC obtained using the *K. sucrofermentans* B-11267 strain under static culture on the medium with a molasses concentration of 50 g/L reflect the application prospects of BC as a conduit in regenerative biomedicine. Analysis of its morphology using SEM and AFM enabled us to reveal the three-dimensional mesh structure composed of thin fibers, ranging from 60 to 190 nm in diameter. This nanostructure exhibits high porosity and provides an intensive nutrient metabolism and high oxygen permeability, along with the removal of metabolic products or exudates. These are crucially important for conduits used in vivo.

Thus, the properties of BC-based conduits depend on the strain, medium composition, and culture technique; moreover, static conditions provide minimal structure changes that make them preferable for obtaining biomaterials. IR-spectra showed characteristic absorption peaks (e.g., 3350 cm^−1^ for O-H and 1068 cm^−1^ for C-O-C) indicating cellulose I with a dominant phase I_α_. This was found to be related to the bacterial nature of biosynthesis. The X-ray photograph obtained using the diffractometer showed three main peaks (2θ = 14.4°, 16.8°, 22.5°) corresponding to the crystallographic planes (100), (010), and (110); the crystallinity index was 79.7%. High crystallinity characteristics explain the resistance of BC-based conduits to degradation by enzymes; it is significant for long-term in vivo application, since densely packed crystal regions restrict access for enzymes. However, the orientation of cellulose tapes parallel to the film surface revealed by uniplanarity analysis can influence anisotropic mechanical properties; further study is required in order to optimize the conduits.

The physico-mechanical characteristics of BC-based conduits demonstrate their domination over many similar materials. Tensile stretching up to 57.5%, tensile strength up to 3.608 N, and Young’s modulus up to 8.952 MPa are indicators of high elasticity and strength, meeting the necessary requirements for conduits to provide stable fixation and flexibility in long-term usage. The value distribution (e.g., the average thickness 0.617 ± 0.083 mm and Young’s modulus 7.142 ± 0.776 MPa) reflects the variability associated with structural inhomogeneity caused by biosynthesis peculiarities. It emphasizes the necessity for the standardization of a manufacturing process in order to achieve reproducible characteristics.

The key aspect of the study was the oxygen permeability assessment, since oxygen deficiency can result in apoptotic and necrotic processes. Using an oximeter, we recorded an oxygen permeability value equal to 45.8 ± 0.01% (4.07 ± 0.09 mg/L). This was lower compared to the existing analogues [36], and can be increased as a result of various chemical compounds, although it is sufficient for normal physiological processes. According to data from the literature, a significant increase in porosity can be achieved through partial rupture of the fibrils with exposure to solutions comprising strong alkalis and acids [37]. In addition, the MTT assay showed that the hydrogel based on BC conduits had no significant cytotoxic effect on human embryonic lung cells: the cell survival rate was 92%.

Thus, the findings confirm BC to have unique properties necessary for developing the conduits on its base.

## 4. Conclusions

The present investigation using the *K. Sucrofermentans* B-11267 strain concerns the obtaining and study of BC-based conduit samples designed to be used in regenerative medicine to recover damaged cell structures. BC-based conduits were found to have a unique nanofibrillar structure with a high degree of crystallinity (79.7%), providing strength (Young’s modulus up to 8.952 MPa, tensile up to 57.5%) and resistance to degradation. The oxygen permeability value equal to 4.07 ± 0.09 mg/L demonstrates their capability to support cell metabolism, which is vital for the regenerative processes of tissues and organs. The findings suggest the high biocompatibility and mechanical strength of BC-based conduits, surpassing many analogues compared to the studied characteristics.

Using different modifications of bacterial cellulose, we are planning to further produce tubular structures of various diameters which exhibit improved oxygen permeability and physico-mechanical characteristics, and comprehensively assess the biocompatibility of obtained conduits when they are transplanted to replace damaged nerve regions.

## Figures and Tables

**Table 1:** The values of physico-mechanical parameters of the obtained BC-based conduits.

No	Thickness, mm	Tensile, %	Tensile Strength, N	Young’s Modulus, MPa
1	0.709	43.50	2.761	8.952
2	0.506	43.50	1.879	8.537
3	0.867	53.10	3.608	7.837
4	0.546	38.40	1.591	7.588
5	0.692	46.50	2.432	7.558
6	0.704	47.50	2.486	7.434
7	0.55	30.10	1.163	7.025
8	0.692	43.50	2.005	6.661
9	0.534	47.5	1.585	6.249
10	0.546	57.5	1.639	5.221
M ± m	0.617 ± 0.083	43.8 ± 5.4	1.918 ± 0.514	7.147 ± 0.777

**Table 2:** BC oxygen permeability values.

No	Oxygen Permeability of BC-Based Conduits	
	%	mg/L
1	44.4	3.95
2	44.6	3.95
3	44.6	3.97
4	44.8	3.97
5	45.4	3.98
6	46.4	4.15
7	46.4	4.15
8	47.1	4.20
9	47.2	4.23
10	47.2	4.22
M ± m	45.8 ± 0.01	4.07 ± 0.09

## Data Availability

Sequence data are available from GenBank, NCBI.
